# Unlocking the Value of White Blood Cells for Heart Failure Diagnosis

**DOI:** 10.1007/s12265-020-10007-6

**Published:** 2020-05-04

**Authors:** Stefan Meier, Michiel Henkens, Stephane Heymans, Emma Louise Robinson

**Affiliations:** 1 Faculty of Science and Engineering, Maastricht University, 6211 KR Maastricht, The Netherlands; 2 Department of Cardiology, Cardiovascular Research Institute Maastricht, Maastricht University, 6229 ER Maastricht, The Netherlands; 3 Department of Cardiology, Maastricht University Medical Centre, 6229 HX Maastricht, The Netherlands; 4 Centre for Molecular and Vascular Biology (CMVB), Department of Cardiovascular Sciences, KU Leuven, B3000 Leuven, Belgium

**Keywords:** Heart failure, Biomarker, Diagnosis, Endomyocardial biopsy, White blood cells, PBMCs, Buffy coat

## Abstract

Cardiovascular disease (CVD) is the single greatest cause of mortality and morbidity worldwide. Inciting 85% of CVD fatalities is heart failure, often resulting in or from a myocardial infarction. Early detection along with pharmacological treatment and lifestyle adaptation can result in better prognosis. Biomarkers are molecular or physiological measures that indicate disease presence, status, and severity. However, not all forms of heart failure are created equal. Current mainstay biomarkers for heart failure, including NT-pro-BNP and ejection fraction, lack sensitivity for many patients. Circulating white blood cells and peripheral blood mononuclear cells (PBMCs) are emerging as surrogate biopsies, reflecting molecular changes in the heart. We discuss the advantages of PBMCs over other sources, as well as limitations and considerations. We urge medical center biobanks to collect, isolate and store circulating white blood cells as a rich source of biomarkers to catalyze the discovery of novel diagnostic tools for heart failure.

**Electronic supplementary material:**

The online version of this article (10.1007/s12265-020-10007-6) contains supplementary material, which is available to authorized users.

## Brief Introduction

Heart failure (HF) is a complex heterogeneous syndrome whereby cardiac function is compromised due to an inability of the chambers of the heart to either pump during systole, or fill up effectively during diastole. The result is a reduction in cardiac output (the volume of blood pumped out the heart per minute), with cardiovascular demands of the head and body no longer met. Signs and symptoms of HF include but are not limited to dyspnea, chest pain and tissue swelling (edema).

HF affects approximately 2% of the adult population, which rises to close to 15% in those over 70 years of age. [1]. With the global aging population, the number of persons aged over 60 is expected to increase by 56% between 2015 and 2030, projecting HF to be an even greater socioeconomic burden in a decades’ time without advances in diagnostics and interventions (United Nations statistics 2015).

Not all forms of HF are created equal. Different etiologies and phenotypes of HF have differential prognosis and optimal treatment options, and exhibit variability in sensitivity of current diagnostic tools. Circulating NT-pro-BNP levels and left ventricular ejection fraction measured by echocardiography are the mainstay HF diagnostic tools, and show inconsistent sensitivity and specificity according to the form of HF.

We now know that heart failure with preserved ejection fraction (HFpEF) accounts for ≥ 50% of all HF patients and the prevalence of HFpEF is rising by 1% per year relative to heart failure with reduced ejection fraction (HFrEF). [2].

The underlying pathophysiology of HFpEF is thought to include comorbidity-associated systemic inflammation. Reliable and consistently valid biomarkers are lacking for early and specific diagnosis of HFpEF. Up to 30% of HFpEF patients have “normal” BNP levels (< 100 pg/mL) [3]. Other etiologies of HF also involve significant systemic and tissue inflammation including myocarditis and inflammatory cardiomyopathy. Idiopathic or genetic dilated cardiomyopathy (DCM), such as conditions caused by pathogenic mutations in sarcomeric proteins, also has elevated myocardial macrophage infiltration as well as circulating pro-inflammatory cytokines [4].

A considerable proportion of research resources is dedicated to the search for novel biomarkers for particular HF phenotypes that could be used in place or in combination with the current tools to improve sensitivity and specificity. These include molecular circulating biomarkers such as proteins, RNAs, and metabolites as well as clinical physiological parameters such as risk factors and myocardial strain [5]. The overwhelming majority of promising molecular biomarkers are identified in plasma or serum. However, not a single nor a combination of circulating species of protein, RNA, or metabolite has consistently provided improved sensitivity and specificity for HF diagnosis compared with BNP in external validation and real patient cohorts.

With the aim of tailoring the treatment course and follow-up examinations according to the specific HF diagnosis, prognosis, and molecular pathophysiology of an individual patient, novel and specific biomarker discovery is a move towards personalized health care.

We propose circulating WBCs as the next generation source of molecular biomarkers. This paper explores the current literature, advantages, and considerations in using WBCs as an attainable source of biomarkers for diagnosis of HF.

## Biomarker Sources

The original definition of a “biomarker” is a “cellular, biochemical or molecular alterations that are measurable in biological media such as human tissues, cells, or fluids” [6]. This definition now includes clinical parameters permitted by advanced biological imaging techniques.

### Endomyocardial Biopsy

Endomyocardial biopsies (EMBs) have been used since the mid-twentieth century for the diagnosis of cardiac pathologies [7, 8]. EMBs are obtained through an invasive—mostly fluoroscopy-guided—procedure removing tiny cardiac samples from the right or left ventricle. From the EMB, histological analysis to measure inflammation, fibrosis, and cardiac myocyte hypertrophy can be performed along with PCR analysis to determine the viral load. Complication rates of EMBs are rare (< 0.5–1%) especially when EMBs are obtained from the right ventricular (RV) septum and performed by an experienced cardiologists [9]. While the use of EMBs is often crucial for the detection of specific cardiac diseases like giant cell myocarditis and in the monitoring of cardiac allograft rejection, current indications and guidelines are primarily based on expert opinion, and therefore, the procedures are only performed in few expert centers—with the availability of multi-disciplinary team—around the world [10].

Increasing diagnostic yield should go hand in hand with EMB-guided management that affects the outcome of a subgroup of patients. In DCM—which has an estimated prevalence of > 1:250—EMB-guided management is already used in EMB proven chronic cardiac inflammation, where the positive effect of immunosuppressive therapy on top of guideline recommended heart-failure medication has been shown [11].

There is an urgent need for multi-center trials performed by these expert centers to really show the additional diagnostic and prognostic value of and EMB-guided management strategies. A further drawback is that a diagnosis can be difficult to establish if the needle puncture misses the diseased part of heart muscle. Moreover, since EMBs are only performed in a few centers worldwide there is an urgent need to identify effective and easier to obtain biomarkers for HF pathophysiologies. [12].

### Cell-Free Blood-Based Biomarkers

The primary source of biomarker for HF is blood. Blood is routinely extracted from patients on outpatient and inpatient admission. The extraction of blood has minimal risks when performed by a trained technician. A wide range of health care professions can perform blood withdrawal including biomedical scientists, paramedics, phlebotomists, and nursing staff. Venous blood is nearly exclusively taken from human patients, from the superficial veins of the upper limb.

Whole blood in the clinical is collected in Vacutainer® (BD) blood collection tubes, which are made of sterile glass plastic with a rubber stopper that creates a vacuum seal. This allows clean and precise withdrawal of a set volume of blood. A variety of types of Vacutainer® are available, which contain different additives and coating to optimize according to storage of blood and later analysis.

Ethylenediaminetetraacetic acid (EDTA) and sodium citrate are calcium chelating agents that prevent the blood from clotting. Vacutainers pre-coated with ETDA or sodium citrate are commonly used in the clinic. In this way, the blood can be spun resulting in an upper cell–free liquid layer of blood, the plasma, which also contains coagulation factors. Vacutainer tubes are coated with EDTA or sodium citrate for blood collection for plasma isolation. Serum is a similar cell-free liquid element of blood but after the blood has been allowed to clot at room temperature for 15–30 min, with the blood clot being sedimented and removed by centrifugation.

Other specialized tubes are designed for preservation of specific types of biological molecule, such as nucleic acids, and include PAXgene Blood RNA Tubes (Qiagen) and Tempus Blood RNA Tubes (Life Technologies). These systems are optimized and validated for preservation of intracellular RNAs [13]. Stabilizing extracellular RNAs, such as in plasma and serum, is trickier and more variable depending on the nature of the RNA (length, modifications, associated proteins or contained within extracellular vesicles). With the growth in RNA-based biomarker investigation from plasma or serum samples, new technologies are being developed for preservation of extracellular, cell-free RNA [14, 15].

Plasma and serum are the mainstay source of circulating biomarkers including proteins, non-coding RNAs (microRNAs and long non-coding RNAs), messenger RNAs, and metabolites [16]. However, as RNAs are not actually synthesized in the plasma/serum but are released from cells from the blood and vasculature or from distant tissues, levels of these biomarkers are often low. This presents a problem for detection and sensitivity of assays. Quality of the extracted cell-free RNA, for example, needs to be of high quality to be able to effectively detect low abundance intact markers, with quality sometimes compromised in clinical samples. Not only are the disease-associated markers low in abundance, but normalization methods for total RNA levels is complex. Normalization to total RNA content or the use of specific reference or “housekeeping” genes suffers a serious lack of standardized methodologies across the discipline of translational cardiovascular research [17, 18].

Another confounding factor and consideration in using plasma or serum as a source of RNA-base marker is the negative impact of heparin administration to the patient on RNA abundance, quality and interference of PCR reactions [19, 20].

### White Blood Cells

White blood cells are the main cellular component of blood. WBCs have all conventional cell organelles including a nucleus and mitochondria, with endogenous transcription (RNA, transcriptome), translation (proteome, protein), and metabolic processes such as oxidative phosphorylation occurring within the cells. Through epigenetic mechanisms, they are sensitive to metabolic and biochemical environment. In this way, they may better reflect the transcriptional landscape of distant organs and tissues, acting as an effective liquid, accessible biopsy [21].

With inflammation as a central process in HF initiation or progression, using circulating WBCs as a source of biological marker to specifically diagnose HF may be more appropriate than using simply the fluid layer of blood that contains all cell-free or tissue-secreted content [22].

It has recently come to light that circulating WBCs reflect the intra-cardiac metabolic and transcriptomic status to an unexpectedly high degree.

White blood cells (WBCs), also called leukocytes, are cells of the immune system that are found in the blood, lymphatic system as well as within tissues (macrophages). The role of the immune system is to fight infection and destroy foreign bodies. WBCs are derived from multipotent hematopoietic cells in bone marrow and T cells mature in the thymus. The major categories and sub-categories of WBC have different biological functions. Classifications and roles of WBCs as well as some isolation strategies from whole blood are described in Table 1.

**Table 1:**
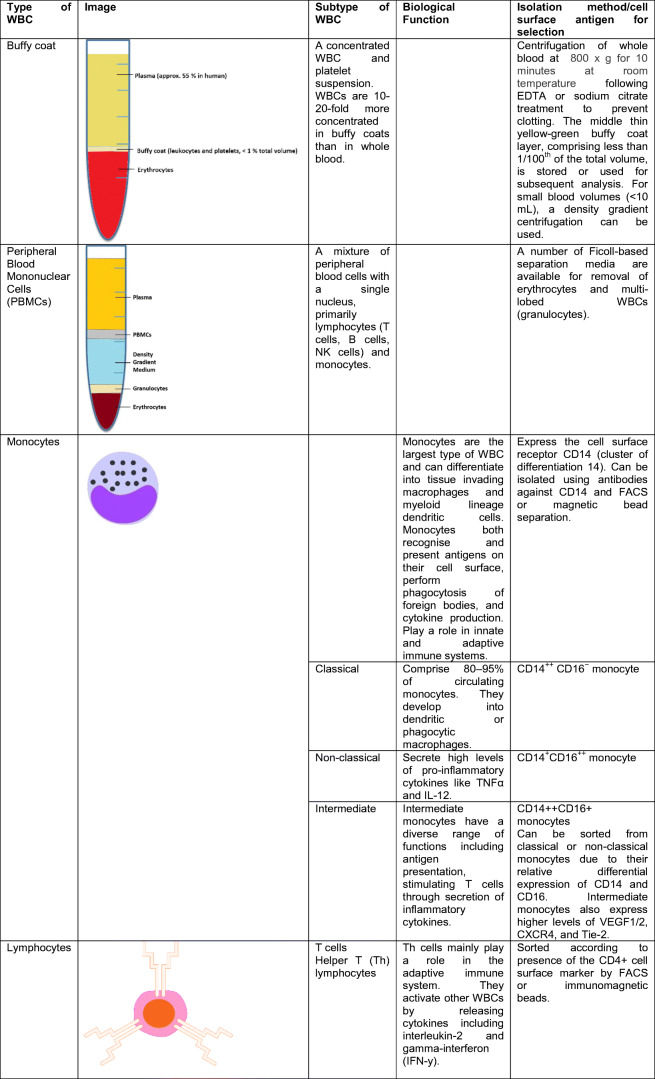

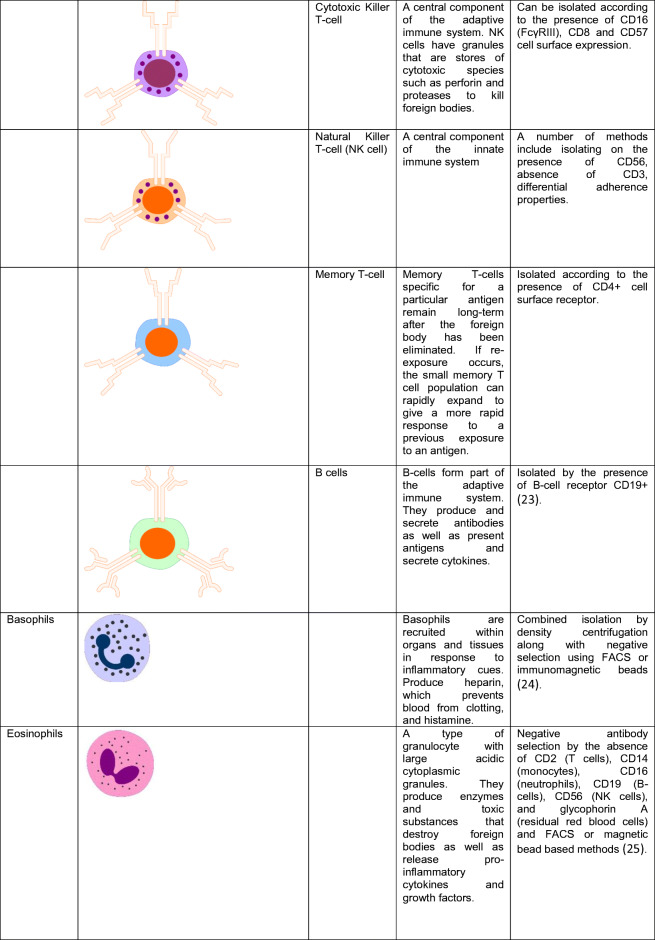
Types of white blood cell in human blood

The buffy coat, the intermediate yellow-green–colored layer between the plasma and red blood cell layer that constitutes about 1% of the total blood volume, contains leukocytes and platelets.. Many biobanks collect and store patient buffy coats in addition for subsequent genomic DNA isolation for genetic analysis, as the plasma or serum is cell-free and the red blood cells are anucleated. Genetic analysis in the HF clinic is often performed for suspected familial DCM patients for classic well-characterized mutations in heart muscle genes such as Titin, Myosin Heavy Chain 7, and Myosin Binding Protein C3.

Peripheral blood mononuclear cells (PBMCs) are a mix of all single nuclear blood cells, namely lymphocytes and monocytes. Lymphocytes and monocytes (the two main cell types that compose PBMCs) can be separated through a pre-plating step. Monocytes adhere to the base of plastic flasks within 2–3 h whereas lymphocytes will remain in suspension. PBMCs are also a source of DNA for genetic analysis, in vitro culturing and functional assays or for subsequent isolation of lymphocyte or monocyte sub-types, provided appropriate cryo-preservation. PBMCs are emerging as a rich source of circulating molecular biomarker in CVD.

## The WBC Transcriptome May Act as a Surrogate for the Myocardial Transcriptome

Myocardial tissue is comprised primarily of cardiac myocytes, cardiac fibroblasts, and endothelial cells along with cells of the vasculature such as smooth muscle cells and pericytes [23]. Heart muscle also contains resident macrophages even in the healthy individual, which increases considerably when cardiac injury or disease ensues [24–26].

Surprisingly, considering WBCs comprise a relatively small proportion of the total cellular population of the heart, a number of studies have found that the RNA, protein, and metabolite profiles of WBCs reflect that in heart tissue from the same individual (human or animal). In this way, WBCs are considered a surrogate liquid biopsy.

Wider studies examining the relationship between the peripheral blood transcriptome and nine human tissues, showed a strong correlation (> 80%) for all tissues tested. Only the spleen and stomach showed a closer relationship with that of the peripheral blood than the heart (84.2%) [27].

Some HF biomarker investigation studies, though limited in number, have utilized WBCs, predominantly in the form of isolated PBMCs. Table 2 presents a reference table to these published studies.

**Table 2:** White blood cell-based biomarkers for heart failure

Source biomarker	Species	Heart failure phenotype	Biomarker findings	References
Whole blood leukocytes	Human	Severe heart failure (NYHA III-IV)	mRNA:Na/Ca exchanger (NCX) was elevated in WBCs from HF patients compared with heathy controls, as well as in myocardial tissue from HF patients.	[28]
Mononuclear blood cells	Human	Asymptomatic left ventricular dysfunction (ALVD) or pre-heart failure	NGFB, TMEM79 and FBXW7 down-regulated in ALVD. FECH, ALK, UBN1, and SLC43A2 increased in ALVD.	[29]
PBMCs	Rat	Hypertensive Heart Disease (HHD)	Overall commonalities between PBMC and cardiac metabolism and transcriptome. [Ca2+]i, [Zn2+]i and 8-isoprotane and increased H_2_O_2_ production in cardiac mitochondria as well as PBMCs, indicating oxidative stress in both. Pathway analysis revealed elevated gene expression similarly in both the heart and PBMCs involved in p38MAPK, NFκB, and TGF-β1 signaling in HHD.	[21]
PBMCs	Human	Chronic heart failure (CHF) patients: ischemic (ICM) and non-ischemic DCM (NIDCM).	MicroRNA-107, −139, and − 142-5p downregulated in CHF patients versus control subjects. MiR-142-3p and -29b upregulated in NIDCM patients. MiR-125b and − 497 downregulated in ICM patients.	[30]
PBMCs	Human	HFpEF	miR-26b, miR-208b, and miR-499 were elevated in hypertensive HFpEF patient PBMCs.	[31]
PBMCs	Human	Chronic heart failure	Mitochondrial transmembrane potential (MTP) and reactive oxygen species (ROS) levels in PBMCs correlated with serum NT-proBNP and short-term prognosis in CHF patients.	[32]
PBMCs	Human	Compensated and decompensated heart failure	SIRT1 mRNA expression downregulated in patients with both compensated and decompensated heart failure.	.. [33]
PBMCs	Human	Congestive heart failure	CHF patient PBMCs show structural and functional dysarray of mitochondria with elevated reactive oxidant species production.	[34]
PBMCs	Human	Heart failure with left ventricular assist device (LVAD)	Levels of reactive oxygen species (ROS) and oxidized low density lipoproteins (oxLDL) were elevated in HF patients with LVAD than baseline or control group. PBMCs from LVAD recipients had higher γ-H2AX foci (DNA damage) and double-strand DNA breaks and superoxide dismutase (SOD).	[35]
PBMCs (along with cardiac biopsies)	Human	HF	CDKN2B-AS1/ANRIL upregulated and HOTAIR and LOC285194/TUSC7 downregulated in PBMCs similarly to in LV tissue in HF patients.	[36]
PBMCs	Rat	Doxorubicin-induced toxic cardiomyopathy	Similar overall transcriptome changes between the heart and PBMCs, 2411 genes similarly differentially regulated. Pathway analysis revealed a correlation between upregulation of genes involved in the oxidative stress response and protein ubiquination.	[37]
PBMCs	Human	Heart failure NYHA classification II-III	Differential abundance of the S-nitrosylated proteome in PBMCs was assessed. 93 differentially expressed and 111 differentially S-nitrosylated proteins were identified in HF patients compared with healthy controls. ATP synthase, thrombospondin-1 (THBS1), and vinculin (VCL) were the top differentially abundant and S-NO-modified proteins.	[38]
PBMCs	Human	Chagasic cardiomyopathy. *Trypanosoma cruzi* (Tc) causes Chagas cardiomyopathy.	213 and 199 proteins were differentially abundant in asymptomatic and symptomatic compared to healthy controls. A panel of MYC/SP1 transcription factors that regulate hypoxia and oxidative/inflammatory stress (MYC, SP1, MYCN, and ANGPT2) were associated with disease progression in Tc + subjects.	[39]
PBMCs	Human	Chronic heart failure	18 proteins were differentially expressed between controls and CHF patients. 11 of which (CAPZA2, CCT2, TLN1, TCTP, YWHAZ, EEF1D, NME1, NDUFS1, PRP19/PSO4, HSP27, TCP-1-epsilon) fell into 4 functional classes: 3 cytoskeletal, 4 cell-cycle progression, 2 stress response and DNA repair, 2 energetic metabolism proteins.	[40]
PBMCs	Human	Chronic heart failure of varying degrees of severity (NYHA I-IV)	PBMCs from CHF patients had increased TNFα and IL-6 RNA and elevated TNFα and IL-6 protein secretion from PBMCs in culture. f TNFα and IL-6 positively correlated with severity of disease (lower ejection fraction or higher NYHA class) and were also higher in CHF of ischemic etiology rather than non-ischemic.	[41]
PBMCs	Human	Coronary heart disease (CHD) patients ± different etiologies of HF	Interleukin-37 mRNA levels in PBMCs higher in CHD than healthy controls but even higher (4-fold compared with 2.5 fold) in acute myocardial infarction HF patients.	[42]

## Methodology and Pre-analytical Variables

In biomarker studies, measuring, analyzing, documenting, and communicating the flow and timing of taking blood-based samples are keys to comparing and replicating biomarker investigations. The source of WBCs, as well as cell-free markers, could be a confounding factor for analysis and for comparing pre-clinical, translational studies with clinical cohorts. Guidelines and tools are available for collating, storing, and sharing the most important pre-analytical quality parameters of biospecimens used for research. For example, the Sample PREanalytical Code (SPREC) is a regularly updated software tool that allows documentation of sample type, primary container type, pre-centrifugation conditions (time and temperature between collection and processing), detailed isolation method, and long-term storage conditions [43]. Other guidelines include the Minimum Information About BIobank data Sharing (MIABIS) [44]. However, many biobanks have developed their own in-house tools and methodologies for collection of pre-analytical information on biospecimen. The lack of standardization and homogeneity between biobanks on a global scale is a limitation in designing and analyzing results of large-scale multi-center studies, with external validation being a requirement for identification and verification of a novel biomarker.

Predominantly, venous blood is taken from patients from the cubital fossa area of superficial veins close to the skin (inside of the elbow), where a venipuncture is easy and minimally invasive [45]. Arterial blood extraction is possible in a hospital setting but is reserved for measuring arterial blood gas concentrations, rather than biomarkers.

For pre-clinical work designed to identify circulating biomarkers from animal models of disease, the blood is extracted from a range of locations including but not limited to the saphenous vein, tail vein, jugular vein, marginal ear vein/artery, and posterior or inferior vena cava (for terminal procedures). A further terminal procedure for extracting large quantities of good quality blood is a terminal cardiac puncture from a euthanized small animal.

The route of extraction can significantly confound findings and should also be documented. Moreover, methods and routes of blood extraction in pre-clinical models may not reflect that taken from the patient in the clinic, and this should be taken into account in translational research.

As covered in the aforementioned Sample PREanalytical Code, the time of day can affect the composition in blood-derived biospecimen, either due to circadian rhythm fluctuations or effect of food intake time and type. The fluid component of blood carries metabolites such as glucose, lipids, enzymes, hormones, and ions that are highly sensitive to timing of extraction and lifestyle. This is the reason that fasting blood tests are often requested, where the patient must not drink or eat anything other than water for up to 12 h before blood withdrawal. In practice, such as emergency room admission, this is not always possible. Some plasma-based RNAs, metabolites, and solutes show heterogeneity according to the time of day or post-prandially [46–48]. WBCs, being a cellular component with regulated uptake and transmembrane transport, could exhibit less inter- and intra-individual heterogeneity.

In order to examine the effect of the extraction-processing interval, we conducted a pilot study (Supplemental Figure, unpublished). PBMCs were extracted, and a time course was conducted. RNA integrity and relative cell death were examined for extraction-processing intervals between *t* = 0 and *t* = 6 h. During the interval, whole blood was placed on ice in EDTA Vacutainer® tubes and brought to room temperature for 30 min prior to PBMC isolation using SepMate™ PBMC Isolation Tubes and Lymphoprep™ Density gradient medium (Stemcell Technologies). We identified no significant compromise in RNA integrity or relative cell death of PBMCs between 0- and 6-h post-extraction in these conditions.

With the confounding pre-analytical variables considered, for PBMCs and other WBCs to evolve as a mainstay source of biomarkers, certain standardized quality control measurements should be implemented and integrated into the documentation and data collection tools used by biobanks. As per the supplemental figure, cellular RNA integrity can be analyzed by measuring the ribosomal RNA 18S:28S ratio by visualization and quantification on a gel, or sophisticated automated methods such as the Tapestation or the Bioanalyzer (both Agilent). However, these analyses only assess RNA degradation. Changes in the relative abundance and expression of different RNA species (the transcriptome) are yet to be thoroughly investigated for different pre-analytical variables, including the extraction-processing interval, storage method, and freeze-thawing cycles.

Cell death (apoptosis, necrosis) can be analyzed using stains that distinguish live from dead cells, such as propidium iodide or other commercially available live-dead stains followed by microscopy or flow cytometry for quantification of cell death. Other quality measures include verifying activation status of PBMCs. Pre-analytical conditions, such as pre-centrifugation time and temperature and freeze-thawing could cause WBC activation and production of cytokines post-extraction, giving misleading results in biomarker investigations. Cell surface markers specific for activated WBCs, such as CD206 (mannose receptor) as a marker for macrophages that have transitioned from an M1 to M2 phase, can be used to assess the degree of activation. Such protocols to assess activation in PBMC biospecimen populations are yet to be robustly established.

## Conclusion

WBCs are emerging as an important and accessible source of biologically meaningful biomarkers, with more biological information relevant to organ health status than plasma or serum, fulfilling the criteria of a biomarker, “cellular, biochemical or molecular alterations that are measurable in biological media such as human tissues, cells, or fluids.” In particular, the transcriptome of PBMCs has been shown to strongly reflect that of the myocardium in both health and disease, acting as a surrogate liquid biopsy without the need for a highly invasive EMB.

Based on our discussion, medical center biobanks should routinely collect WBCs in the form of PBMCs as well as storing in a manner to preserve RNA, DNA, and protein and enable separation and analysis of WBC types following freeze-thawing. New tubes and kits are being developed for optimal collection and storage of blood cells in a clinical environment.

What’s more, multi-omics systems biology approaches could be combined and implemented to more effectively evaluate the interactions and efficiency of WBC biomarkers in diagnosing and distinguishing different heart disease phenotypes [49].

Whilst our discussion focused on HF, with inflammation playing a central role in other cardiovascular disorders, such as coronary artery disease and atherosclerosis, the circulating WBC transcriptome is also emerging as reflecting other elements of cardiovascular health.

## Electronic supplementary material

ESM 1 (DOCX 222 kb)

## Abbreviations

CVD: Cardiovascular disease
WBC: White blood cell
HF: Heart failure
HFpEF/HErEF: Heart failure with preserved/reduced ejection fraction
DCM: Dilated cardiomyopathy
PBMCs: Peripheral blood mononuclear cells
(NT-pro-)BNP: (N-terminal pro) b-type natriuretic peptide
