# Supplementary material for: Unlocking the Value of White Blood Cells for Heart Failure Diagnosis
Source: J Cardiovasc Transl Res. 2020 May 4;14(1):53–62. doi: 10.1007/s12265-020-10007-6 (PMC7892730; doi:10.1007/s12265-020-10007-6)
Supplement: Supplementary file 1 — (DOCX 222 kb) [file 12265_2020_10007_MOESM1_ESM.docx]

Supplemental Figure: PBMC RNA integrity and cell death is minimal between 0-6 hours’ extraction – processing interval


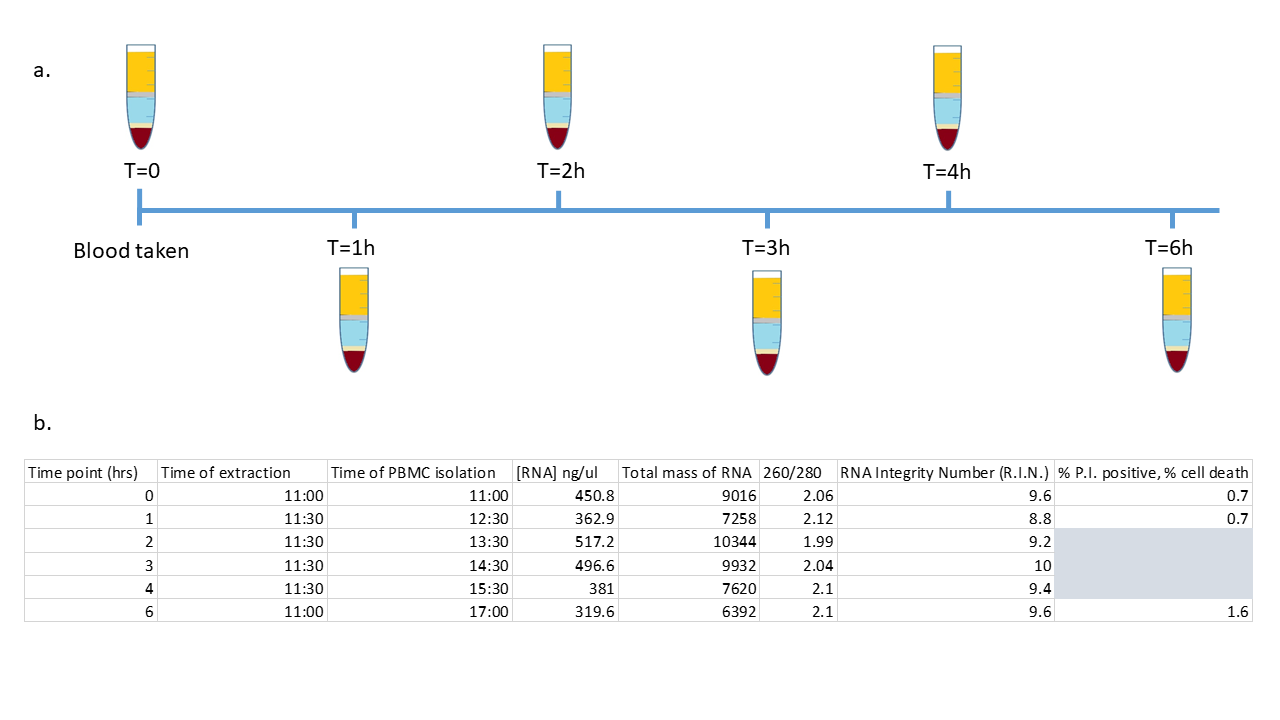

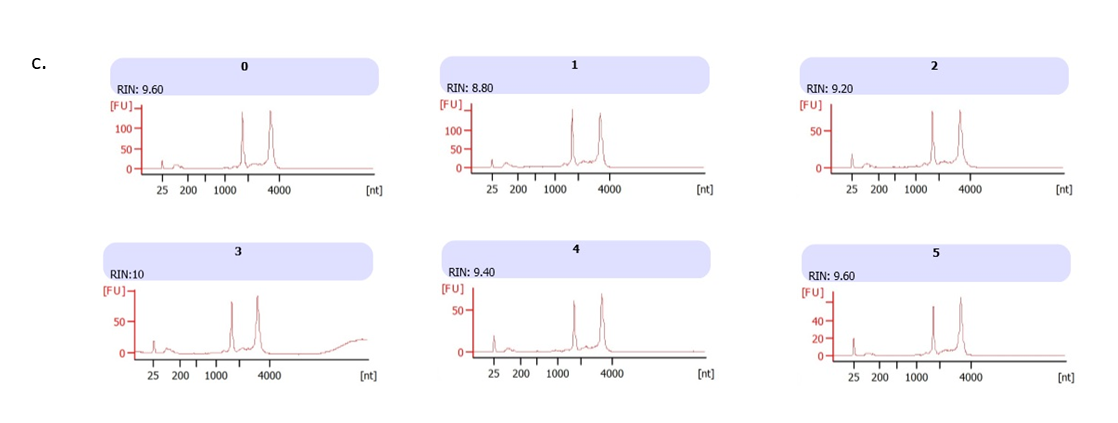


Supplemental Figure: PBMC RNA integrity and cell death is minimal between 0-6 hours’ extraction – processing interval

1. Schematic of experimental set-up
2. Time course data. RNA concentration and purity was analysed using Nanodrop 3000 (Thermo Scientific), R.I.N. was analysed on a Eukaryote Nano RNA chip on a Bioanalyzer 2100 (Agilent) and % cell death by Propidium Iodide (P.I) staining and flow cytometric analysis on a BD Accuri™ C6.
3. Bioanalyzer traces and R.I.N. values derived from Bioanalyzer 2100 measurement. 0=t0, 1=1hr, 2=2hr, 3=3hr, 4=4hr, 5=6hr
